# Nanosponge-Encapsulated Polyoxometalates: Unveiling the Multi-Faceted Potential Against Cancers and Metastases Through Comprehensive Preparation, Characterization, and Computational Exploration

**DOI:** 10.3390/ph18030347

**Published:** 2025-02-28

**Authors:** Muhammad Sajjad, Muhammad Zubair Malik, Ayesha Bint Umar Awan, Hamid Saeed Shah, Muhammad Sarfraz, Faisal Usman, Tahir Ali Chohan, Tanveer A. Wani, Seema Zargar, Zobia Jawad

**Affiliations:** 1 Faculty of Pharmacy, University of Sargodha, Sargodha 40100, Pakistan; sajjadawan121@yahoo.com; 2 Endocrinology and Diabetes, Queens Hospital BHRUT Romford, Romford RM7 0AG, UK; ayesha.awan4@nhs.net; 3 Institute of Pharmaceutical Sciences, University of Veterinary and Animal Sciences, Lahore 54000, Pakistan; tahir.chohan@uvas.edu.pk; 4 College of Pharmacy, Al Ain University, Al Ain 64141, United Arab Emirates; muhammad.sarfraz@aau.ac.ae; 5 Department of Pharmaceutics, Faculty of Pharmacy, Bahauddin Zakariya University, Multan 60800, Pakistan; faisal.usman@bzu.edu.pk; 6 Department of Pharmaceutical Chemistry, College of Pharmacy, King Saud University, P.O. Box 2457, Riyadh 11451, Saudi Arabia; twani@ksu.edu.sa; 7 Department of Biochemistry, College of Science, King Saud University, P.O. Box 22452, Riyadh 11451, Saudi Arabia; szargar@ksu.edu.sa; 8 Lady Willingdon Hospital, King Edward Medical University, Lahore 54000, Pakistan; zobiajawadnaseem@gmail.com

**Keywords:** nanosponges, breast cancer, metastasis, mice, docking

## Abstract

**Background/Objectives**: This study examined the fabrication and characterization of nanosponges (NS) laden with polyoxometalates (TiW_11_Co) with the intention of targeting malignancy. **Methods**: By employing the emulsion solvent diffusion technique, TiW_11_Co-NS were generated by combining polyvinyl alcohol (PVA) and ethyl cellulose (EC) in different concentrations. **Results**: A significant numerical results encompassed a hydrodynamic particle diameter of 109.5 nm, loading efficiencies reaching 85.9%, and zeta potentials varying from −24.91 to −27.08 (mV). Scanning and transmission electron microscopy were employed to validate the TiW_11_Co-NS porous structure and surface morphology. The results of the stability investigation indicated that TiW_11_Co-NS exhibited prolonged sturdiness. Investigation examining the inhibition of enzymes revealed that TiW_11_Co-NS exhibited enhanced effectiveness against TNAP. Pharmacological evaluations of TiW_11_Co-NS demonstrated improved cytotoxicity and apoptotic effects in comparison to pure TiW_11_Co, thereby indicating their potential utility in targeted cancer therapy. In vivo investigations involving mice revealed that TiW_11_Co-NS caused a more substantial reduction in tumor weight and increased survival rates in comparison to pure TiW_11_Co. The resemblance of TiW_11_Co for crucial proteins associated with cancer proliferation was featured through molecular docking, thereby supporting its therapeutic potential. **Conclusions**: The TiW_11_Co-laden nanosponges demonstrated superior stability, enzyme inhibition, cytotoxicity, and in vivo anticancer efficacy, underscoring their potential for targeted cancer therapy.

## 1. Introduction

Cancer is an anthology of problems classified by the proliferation of peculiar cells that undergo unregulated division, resulting in the formation of a unified neoplastic growth represented as a tumor [1]. Normal cells may be disturbed by faults that arise during cell division, leading to genetic mutations. Moreover, exposure to dangerous environmental pollutants can also induce gene mutations [2]. Metastasis pertains to the process by which a tumor has the capacity to distribute and subvert adjacent tissues and anatomical structures [3]. Metastatic malignancies are distinguished by elevated mortality rates and present greater difficulties in terms of medical intervention [4]. Cancer is considered one of the leading causes of death on a global scale. As per the World Health Organization (WHO), cancer accounted for approximately 20% of all documented fatalities in 2023 [5]. The identification and management of cancer present a complex and demanding task given the diverse nature of cancer cells and the intricacies involved in their staging [6].

Polyoxometalates (POMs) refer to clusters composed of inorganic transition metal oxides (namely vanadium, molybdenum, and tungsten) that possess a negative charge. The transition metals at their greatest oxidation state are connected by oxo-ligands (O_2_^−^) inside a three-dimensional framework [7]. The diverse nature of their molecular makeup and features allows for a wide range of applications in the treatment of metabolic illnesses, such as cancer and diabetes, as well as infectious diseases caused by bacteria, viruses, and parasites [8,9,10]. POMs exhibit several limitations, such as their relatively larger dimensions in comparison to other nano-sized compounds used in cancer treatment [11]. Additionally, their integration into cellular structures poses challenges, and their stability under physiological pH conditions is compromised [12,13].

There has been a tremendous increase in research dedicated to nanosponges (NS) and their potential use in personalized drug delivery, which has attracted considerable interest in the scientific community [14,15]. The precision of NS delivery systems in controlling the release rates of medications or guiding drugs to precise cellular sites is anticipated to have a substantial influence on the healthcare system [16]. The nanosized delivery system, e.g., NS, has distinct advantages for drug delivery purposes, owing to its remarkable stability, substantial carrier capacity, and the ability to accommodate both hydrophilic and hydrophobic compounds [17]. The work in this specific field is spurred by the potential of NS for accurate and targeted delivery of pharmaceuticals [15,18]. The objective of this study was to analyze the formation, physical, pharmacological, and biological effects of NS containing TiW_11_Co, which has been investigated for the first occurrence and has never been documented in previous reports.

## 2. Results and Discussion

### 2.1. Characterization and Optimization of TiW_11_Co-NS: A Physical Perspective

A complete comparison of numerous important parameters that might influence the formulation of TiW_11_Co-NS is shown in Table 1. Variations in concentration were applied to each formulation, which were labeled as F1, F2, F3, and F4, to investigate the impact that these factors had on the physical properties of NS.

The percentage of TiW_11_Co (% *w*/*v*) was consistently maintained at 1% *w*/*v* throughout all formulations, demonstrating a steady base concentration for the NS. A clear difference was observed among the formulations in the concentration of EC and PVA in terms of weight percent (*w*/*v*). Both EC and PVA were evaluated at two different concentrations: 50% and 100% *w*/*v*. All formulations were prepared at 10,000 rpm to ensure uniformity in mixing. Characterization results had provided clarity regarding the ways in which these factors affect NS characteristics.

When designing NS, there are two crucial issues that require careful study. Firstly, it is essential that NS possess sufficient size to impede renal excretion [19]. Additionally, it is essential that these entities possess a sufficiently diminutive size to elude phagocytosis and subsequent elimination by the reticuloendothelial system (RES) [20]. The available evidence indicated that biomolecules with molecular weights exceeding 40 kDa and NS ranging from 10 to 500 nm have the ability to infiltrate the capillary network and amass inside the interstitial regions of tumors, enabling the use of passive targeted strategies [21]. Furthermore, a research investigation had provided evidence indicating that the human immune system exhibits a higher level of efficacy in the removal of NS with a size exceeding 200 nm [22]. The analysis of NS size diversity revealed notable disparities across the different formulations, with average NS size ranging from 109.5 nm to 471 nm. The results demonstrated that changes in the concentrations of EC and PVA had an impact on NS size, with lower concentrations of EC resulting in smaller NS. The potential enhancement of NS stabilization in F2 via the increase in PVA concentration might lead to a reduction in NS diameters. This might be attributed to many processes, including greater surface covering, altered nucleation kinetics, and enhanced control of particle growth dynamics. Likewise, it was observed that formulations F3 and F4 exhibited larger NS sizes due to the greater percentage of EC (100% *w*/*v*) and the varying amount of PVA. The elevated viscosity and polymer concentration in F3 and F4 might facilitate the aggregation of NS during the formulation process, leading to an increase in NS sizes. Furthermore, the increased concentration of PVA in F4 (100% *w*/*v*) relative to F3 (50% *w*/*v*) has the potential to improve NS dispersion and reduce their aggregation propensity. Therefore, F4 revealed a somewhat reduced NS size in contrast to F3.

The adaptation in loading efficiency uncovered across formulations F1 to F4 might be attributed to disparities in the composition of the formulations and their related impacts on TiW_11_Co-NS properties. The noticed improvement in loading efficiency from F1 to F2 and from F3 to F4 might be elucidated by the role of PVA as a stabilizing agent and its influence on the encapsulation process. The robust stabilizing impact of PVA might lead to more efficient encapsulation of TiW_11_Co within the NS when utilizing formulations with higher concentrations of PVA (F2 and F4). The elevated concentration of PVA facilitated the creation of additional binding cavities for the TiW_11_Co-NS, hence enhancing their encapsulation and reducing the likelihood of premature release during the formation process. Moreover, the elevated viscosity that had resulted from higher concentrations of EC and PVA (F3 and F4) might also enhance the loading efficiency by reducing the rate of diffusion of TiW_11_Co molecules during encapsulation. Consequently, this might facilitate enhanced entrapment within the NS matrix.

The increased polymer content resulting from higher EC concentrations (F3 and F4) had led to enhanced entrapment efficiency (78.32 ± 6.18% and 85.9 ± 3.21%) due to the formation of a denser and more cohesive matrix. Therefore, this facilitated the effective entrapment of a larger amount of TiW_11_Co. Formulations containing higher concentrations of PVA, specifically F2 and F4, exhibited enhanced entrapment efficiencies for TiW_11_Co. This was attributed to the improved accessibility of PVA molecules, which might promote the development of a more consistent and stable NS structure. Consequently, the percent entrapment efficiencies for F2 and F4 were higher, with values of 82.16 ± 5.91% and 85.9 ± 3.21%, respectively.

Furthermore, variations in the uniformity of NS size distribution might be seen by the examination of polydispersity index (PDI) evaluations; lower PDIs suggest a more limited size range. The observed PDI values, between 0.031 and 0.136, suggested varying degrees of NS size homogeneity. This phenomenon might perhaps be attributed to variations in the quantities of polymers used. The decrease in PDI observed between F1 and F2 indicated that the formulation F2 has a more homogeneous size distribution of nanostructures compared to F1. The observed improvement in NS uniformity may be attributed to the higher concentration of PVA and lower quantity of EC. This might be expected to promote the longevity of the NS, resulting in a narrow size range and a decreased PDI. In terms of PVA and EC content, formulation F4 exhibited the highest levels. The elevated concentration might lead to NS aggregation or an uneven dispersion of NS dimensions. This phenomenon might impede the formation of uniform NS and therefore result in an insufficient stabilization, hence leading to elevated PDI values.

Assessing the zeta potential is necessary for evaluating the charge, stabilization, and dispersion properties of TiW_11_Co-NS. Higher zeta potential levels are indicative of increased stability inside the entire structure, whereas lower values suggest a propensity for colloids to agglomerate [23,24,25]. Zeta potential values ranging from −24.91 mV to −27.08 mV were observed, indicative of surface charge characteristics. The results indicated that changes in the concentrations of EC and PVA had an impact on the surface potential of the TiW_11_Co-NS, thereby influencing their stability and their interactions with biological systems. The PVA had an influence on the surface properties of NS, namely zeta potential, by its interaction with the NS surface and subsequent alteration of the charged group configuration. It is anticipated that formulations F2 and F4, characterized by a greater concentration of PVA, might exhibit an increased quantity of PVA molecules that were readily available for adhesion onto the NS surface. This phenomenon might result in a more pronounced modification of the surface charge. Furthermore, the heightened concentration of PVA in formulation F2 had the potential to influence the aggregation properties and colloidal stability of the NS. The use of PVA molecules as steric stabilizers might be attributed to their ability to form a protective barrier surrounding NS, so impeding their aggregation via the mechanism of steric repulsion. The higher concentration of PVA in formulation F2 had provided enhanced steric stabilization, resulting in a more uniformly distributed and stable colloidal system. This might account for the observed differences in zeta potential between F2 and F1. The significance of spotting a little negative charge on the cell membrane lies in its ability to enhance electrostatic interactions involving the membrane surface as well as positively charged NS [26]. Insights into the preferential accumulation of NS with a small positive charge at the tumor site after systemic injection have been derived from animal studies [27]. The experimental results highlighted the significant influence of different concentrations of EC and PVA on several parameters related to TiW_11_Co-NS, as shown in Table 1.

### 2.2. Scanning Electron Microscopy

**Figure 1:**
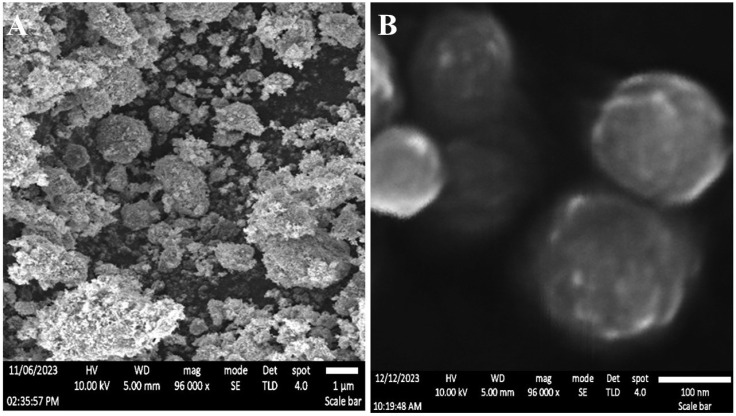
The Scanning electron microscopy (SEM) images of TiW₁₁Co-NS. It displays the morphological characteristics and surface texture of TiW₁₁Co-NS (**A**). It also provides a closer view, emphasizing the TiW₁₁Co-NS architecture and particle distribution (**B**).

The physical characteristics of NS were significantly enhanced with the incorporation of additives. One of the additives that has received a lot of attention for its potential to improve the porous structure of NS is PVA. This indicates that the optimal use of PVA helps create a porous network in the NS matrix (see Figure 1A,B), which might lead to better drug loading, release dynamics, and ultimate NS system performance.

### 2.3. Transmission Electron Microscopy

Transmission electron microscopy (TEM) research was used to assess the surface characteristics of TiW_11_Co-NS. The morphology of the TiW_11_Co-NS, as seen in Figure 1A,B, had a spherical form and smooth, unbroken edges, as evidenced by the absence of any flaws. Previous research on the surface structure of TiW_11_Co-NP at varying TiW_11_Co proportions was consistent with our findings [28]. The homogeneity in the structural features of NS underscored the repeatability and reliability of our findings. The outcomes of our study provided further support for the enhancement of our understanding of the surface characteristics of TiW_11_Co-NS and their possible implications for various applications.

### 2.4. Stability Test of TiW_11_Co-NS

**Figure 2:**
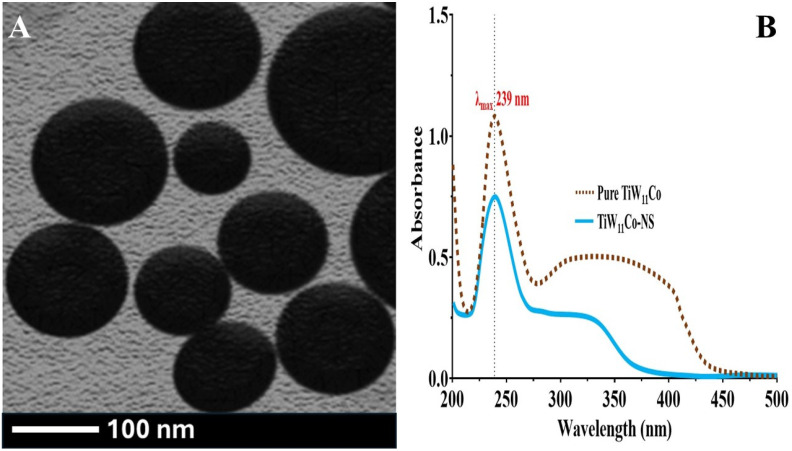
Analysis via transmission electron microscopy (TEM) (**A**). Stability evaluation reveals the λ_max_ of pure TiW_11_Co (depicted by the brown line) and the corresponding wavelength observed in TiW_11_Co-NS after a six-month storage period (illustrated by the cyan line) (**B**).

The findings demonstrated that the stability of TiW_11_Co inside NS remained consistent throughout this study, as seen by the unaltered wavelength (λ_max_) of 239 nm. The confinement of TiW_11_Co inside the NS likely provided protection from environmental factors that might facilitate degradation, such as hydrolysis or oxidation. In addition, the elements of the formulation, namely EC and PVA, did not exhibit any notable associations with TiW_11_Co that might have altered its spectrum properties. This observation provided further evidence that the TiW_11_Co and additives used in the formulation displayed compatibility, hence enhancing the long-term stability of TiW_11_Co-NS (Figure 2B).

### 2.5. TiW_11_Co Release Kinetics

**Figure 3:**
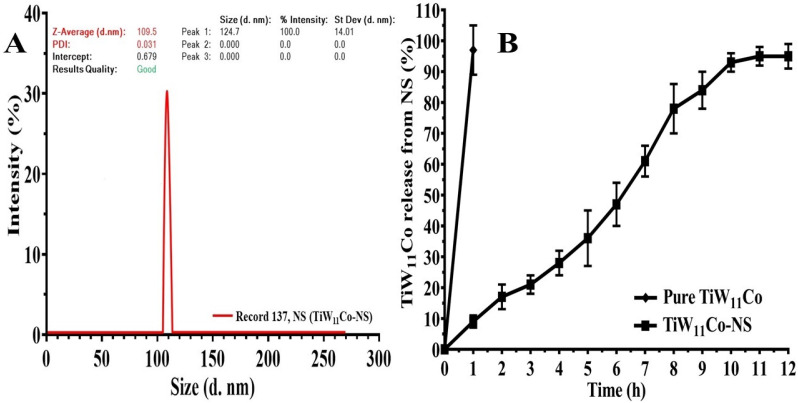
Determining the hydrodynamic diameter of TiW_11_Co-NS (**A**) and assessing its dissolution behavior under pH 7.4 conditions (**B**).

In Figure 3B, the dissolution profiles of both pure TiW_11_Co and TiW_11_Co-NS are depicted. Pure TiW_11_Co was completely dissolved within two hours, although the limited data points posed challenges in constructing a kinetic model. The rapid disintegration of TiW_11_Co might be attributed to its exceedingly short biological half-life [29]. Nevertheless, the release kinetics techniques provided insight into the drug delivery mechanism of TiW_11_Co-NS. Various kinetic models, including zero-order, 1st order, Peppas, and Higuchi equations, were applied to analyze the release characteristics. The R^2^ coefficient served as a quantitative metric for evaluating the kinetics and release patterns of TiW_11_Co from NS [30].

The R^2^ results derived from zero-order analysis (0.9713) implied that the concentration of the TiW_11_Co does not exert influence on its release from NS within a specific time frame. The kinetic model utilized in this investigation illustrated the sustained release properties of TiW_11_Co-NS. The Peppas model displayed a notable R^2^ value (0.9783, n = 0.916), suggesting that the release mechanism of TiW_11_Co from NS involves swelling, erosion, and a non-Fickian super case II type of release [31].

### 2.6. Enzyme Inhibition Studies

**Figure 4:**
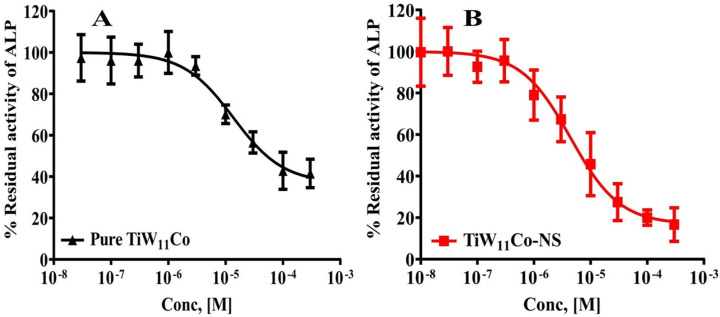
The enzyme (TNAP) inhibition capability of TiW11Co (**A**) and TiW11Co-NS (**B**).

It was found that TiW_11_Co-NS had a substantially stronger inhibitory activity against TNAP in comparison to pure TiW_11_Co. The evidence that supports this assertion is the much lower concentration that is necessary to achieve maximal inhibition. To be more specific, the IC_50_ concentration of TiW_11_Co-NS was found to be 1.87 ± 1.005 µM, which was the highest level of inhibition attained (Figure 4A). On the other hand, pure TiW_11_Co needed a higher concentration of 5.773 ± 2.823 µM to reach a comparable degree of inhibition (see Figure 4B).

The findings of this study indicated that the effectiveness of TiW_11_Co-NS was almost three times more than that of pure TiW_11_Co. This suggested that there might be improvement in bioavailability, solubility, or interactions with the TNAP enzyme. The use of NS could augment the surface area accessible for enzyme interactions, hence possibly resulting in improved binding and inhibitory efficacy.

The enhanced efficacy of TiW_11_Co-NS implied its potential as a more efficacious therapeutic agent for illnesses requiring modulation of TNAP activity. This might include circumstances such as the spread of cancer into bone, whereby TNAP might assume a pivotal function in the processes of mineralization. TiW_11_Co-NS might provide benefits in terms of treatment and enhanced patient compliance by necessitating a lesser dosage to achieve the intended inhibitory effect.

### 2.7. Pharmacological Assessment

#### 2.7.1. SRB Analysis

**Figure 5:**
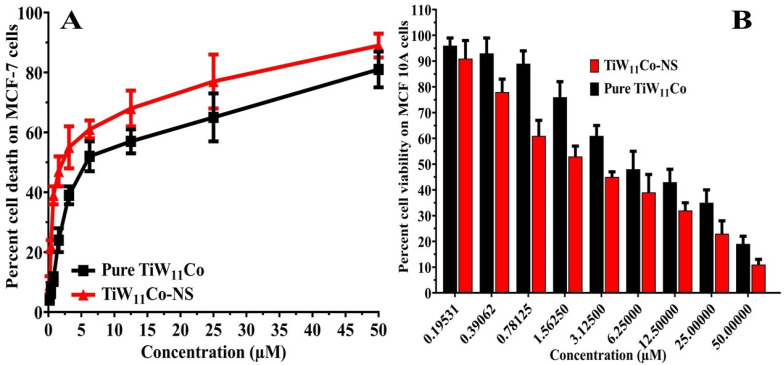
The pharmacological characterization assesses the extent of cell death (**A**) and cell viability (**B**) modulation induced by pure TiW_11_Co and TiW_11_Co-NS on MCF-7 and MCF10A cells, respectively.

Purified TiW_11_Co and TiW_11_Co-NS were tested for their cytotoxic effects on MCF-7 cells using the Sulforhodamine-B (SRB) assay, the results of which were shown in Figure 5A. The MCF-7 cells and MCF-10A normal breast cells (Figure 5B) were exposed to different doses of pure TiW_11_Co and TiW_11_Co-NS. The MCF-7 cell-derived IC_50_ values for pure TiW_11_Co and TiW_11_Co-NS were calculated as 2.45 ± 1.11 and 0.976 ± 0.452 µM, respectively.

Previous studies have shown that pure TiW_11_Co has anticancer effects on many cancer cells, notably KB cells [32] and HCT-116 [33] cell lines, when tested either alone or in combination with chitosan nanoparticles. It is worth mentioning that the composite TiW_11_Co-NS was more effective than pure TiW_11_Co in triggering cytotoxic responses in the cells indicated earlier. This highlights the fact that TiW_11_Co could kill cancer cells on its own and that it may be much more effective when combined with NS. These results highlighted the potential of TiW_11_Co-NS for targeted cancer treatment, paving the way for more research into their mechanisms of action and therapeutic usage.

#### 2.7.2. Method for Evaluating Cell Death Using DAPI Staining

**Figure 6:**
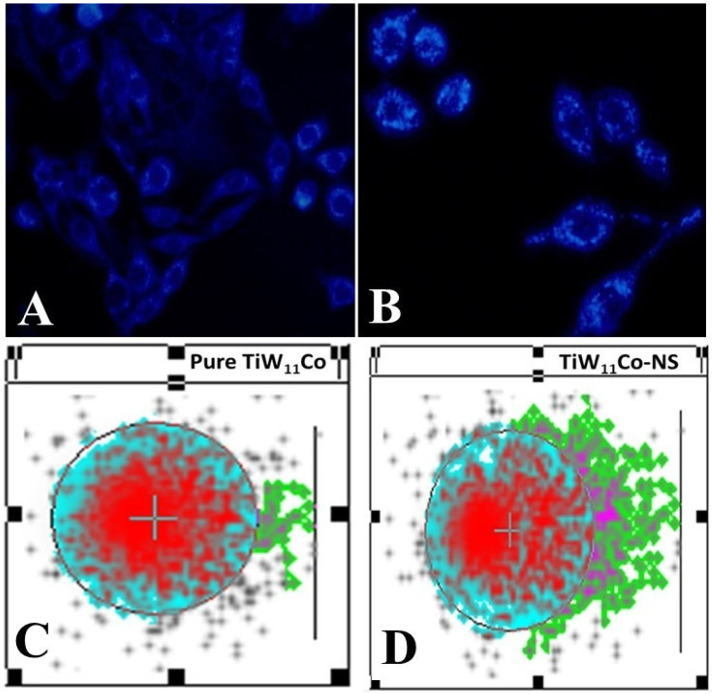
The fluorescence microscopy images (Scale bar set at 10 µm) showing DAPI staining of TiW11Co shows weak nuclear localization, indicating limited cellular interaction (**A**), whereas TiW11Co-NS exhibits enhanced nuclear localization (presence of apoptotic bodies), suggesting improved cellular uptake and hence bioavailability (**B**). The Comet assay analysis of TiW11Co reveals moderate DNA damage, indicating lower therapeutic efficiency (**C**), while TiW11Co-NS shows significantly increased DNA release (long tail DNA), suggesting enhanced cytotoxic and genotoxic potential (**D**).

DAPI staining is an essential technique for qualitative analysis, offering valuable information on changes in nuclear structure, especially valuable for identifying apoptosis [34]. The results obtained from the DAPI labeling of MCF-7 cells treated with pure TiW_11_Co (Figure 6A) and TiW_11_Co-NS (Figure 6B), which indicated the presence of apoptotic bodies with denatured cell membranes, were consistent with previous studies [35,36]. Small alterations in size, shape, and smoothness at the margins were seen in MCF-7 cells when exposed to pure TiW_11_Co, suggesting a very small effect on cellular integrity. In contrast, significant characteristics suggestive of apoptosis were found in MCF-7 cells subjected to TiW_11_Co-NS treatment. The presence of shattered and condensed nuclei in the studied samples indicated the occurrence of chromatin condensation, a distinctive property often associated with apoptotic cells. The observed disparity highlighted the efficacy of TiW_11_Co-NS in promoting apoptosis in MCF-7 cells. The presence of apoptotic bodies, characterized by the ruptured cell membranes and condensed nucleus, indicated the coordinated cellular response to TiW_11_Co-NS therapy. The observed morphological alterations align with prior research, so bolstering the dependability and replicability of our results [37,38].

DAPI staining was used to reveal the breakdown and condensation of nuclei, which served as an indicator of significant cellular processes linked to apoptosis, including the destruction of DNA and nuclear collapse. The observed changes might be caused by the induction of apoptotic pathways induced by TiW_11_Co-NS, which initiated intracellular signaling cascades leading to cellular demise.

#### 2.7.3. Genotoxicity Analysis

The comet assay results, which validate DNA damage in MCF-7 cell lines that were exposed to both free TiW_11_Co and TiW_11_Co-NS, offer significant insights into the possible genotoxic consequences of these substances. Utilizing a highly sensitive method, the comet assay is employed to assess DNA damage at the cellular level. The procedure involved exposing cells to electrophoresis, which triggered the escape of fragmented DNA from the nucleus in the form of a tail resembling a comet. The quantification of DNA damage commonly employed parameters including tail moment (TM) and olive tail moment (OTM), wherein greater values signify augmented damage.

The assessment of DNA damage could be achieved through the comparison of tail DNA to total tail DNA and the evaluation of olive tail moment (OTM) and tail moment (TM). The distal portion of the DNA strand had been significantly impacted by the travel of damaged fragments of DNA away from the nucleus, as indicated by these parameters.

Figure 6C,D depicted the outcomes of the comet test conducted on cells that were treated with purified TiW_11_Co and TiW_11_Co-NS, respectively. The visual depictions provided an indication of the magnitude of DNA damage caused by each compound, with longer and more conspicuous tails denoting a more severe degree of damage.

The quantitative results of various parameters evaluated in the comet assay, such as tail DNA percentage, OTM, and TM, were estimated in Table 2. The presented information gave a comprehensive analysis of the genotoxic impacts inflicted by pure TiW_11_Co and TiW_11_Co-NS on MCF-7 cells.

The genotoxic effects of both purified TiW_11_Co and TiW_11_Co-NS on MCF-7 cells were evident through the detection of DNA damage, as measured by an increased proportion of TM, OTM, or tail DNA, as detailed in Table 2. The comet assay observed enhanced mobility in the distal segment of the DNA strand, which had incurred the most significant damage because of DNA breaks or lesions. An increased level of DNA damage induced by TiW11Co-NS in comparison to pure TiW_11_Co could potentially signify improved cellular uptake or interaction with DNA due to the nanostructured configuration, thereby leading to heightened genotoxic consequences [39,40].

#### 2.7.4. Flow Cytometry Investigation

Flow cytometry is an effective method for studying population-level cellular properties such as size, complexity, and DNA concentration. Using flow cytometry, this work uncovered how the substances pure TiW_11_Co and TiW_11_Co-NS triggered cell cycle arrest [41].

**Figure 7:**
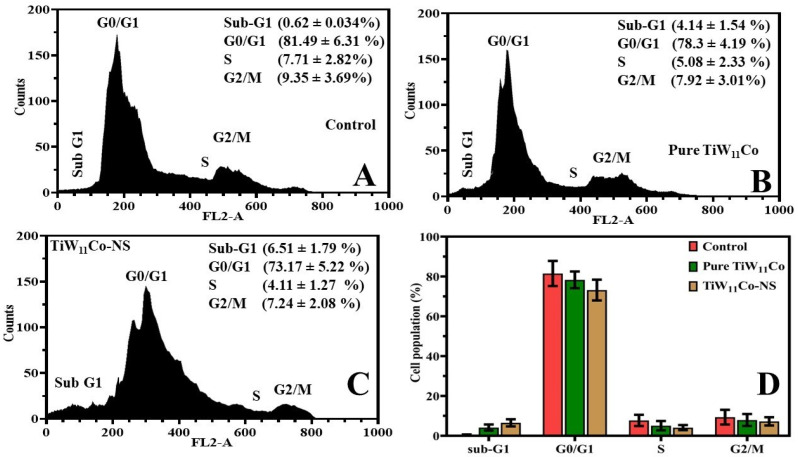
Analysis of MCF7 cells treated with control (**A**), purified TiW_11_Co (**B**), and TiW_11_Co-NS (**C**) via flow cytometry. The proportion of cells that underwent treatment with TiW_11_Co-NS, pure TiW_11_Co, or a control during the cell cycle (**D**).

The administration of pure TiW_11_Co had led to a larger percentage of MCF-7 cells being maintained in the G0/G1 phase of the cell cycle in comparison to the control drug (Figure 7A,B). This observation suggested a possible deceleration in the advancement of the cell cycle. Nevertheless, there was a notable rise in the proportion of cells exhibiting the sub-G1 phase, a characteristic that was often associated with apoptotic or deceased cells. This observation had implied that some cells were halted in the G0/G1 phase, whereas others experienced apoptosis, resulting in cellular demise. The use of TiW_11_Co-NS resulted in a subsequent augmentation in cellular demise in comparison to pure TiW_11_Co (Figure 7C). This is supported by a higher proportion of cells remaining in the sub-G1 phase and a smaller proportion of cells remaining in the G0/G1 phase (Figure 7D). The observed elevated mortality rate associated with TiW_11_Co-NS implied that the nanostructured variant of the drug might augment its cytotoxic properties or cellular absorption, resulting in heightened apoptosis and cellular death [42].

### 2.8. In Vivo Studies

It described an in vivo study conducted on female albino mice to corroborate findings obtained from in vitro experiments regarding the efficacy of various treatments, such as cisplatin, pure TiW_11_Co, and TiW_11_Co-NS [43].

**Figure 8:**
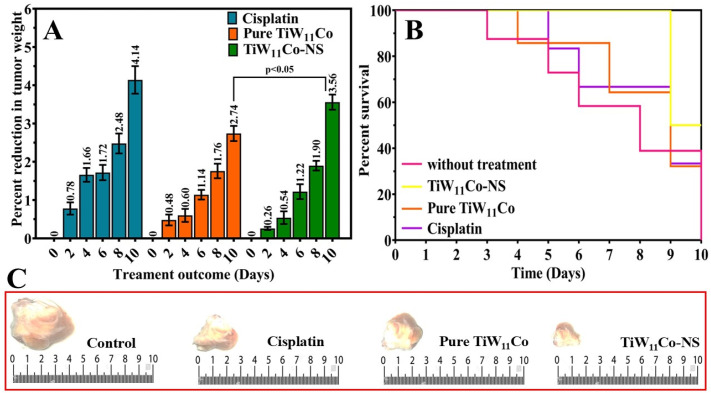
This figure depicts the anticancer efficacy of test compounds on a mice-induced breast cancer model. The results show TiW_11_Co-NS has a significant anticancer effect, lowering tumor size (**A**) and improving survival in treated mice (**B**). The images indicate tumor sizes in different groups in which the TiW_11_Co-NS group produced significantly smaller tumor sizes than those in the other groups (**C**).

On the tenth day following administration, the comparative impact on tumor weight reduction was evaluated between mice treated with TiW_11_Co-NS and those receiving pure TiW_11_Co alone. When comparing pure TiW_11_Co to the other group treated with TiW_11_Co-NS, it demonstrated a more pronounced reduction in tumor weight percentage, suggesting an improved effectiveness in suppressing tumor growth (Figure 8A). This implies that TiW_11_Co-NS might exert a more potent cytotoxic influence on tumor cells, thereby facilitating a more efficient inhibition of tumor growth [44].

This study evaluated and compared the survival rates of mice subjected to specified dosages of pure TiW_11_Co and TiW_11_Co-NS in comparison to the reference standard of cisplatin. The survival rate of mice treated with TiW_11_Co-NS at a dose of 1.756 µM/kg was found to be greater in comparison to those treated with pure TiW_11_Co alone, as seen in Figure 8B. It has been shown in our findings that the tumor-bearing mice that were treated with TiW_11_Co-NS displayed the smallest tumor size compared to all other treatment groups represented in Figure 8C. This recommends that the TiW_11_Co-NS were highly efficient in lowering tumor growth. These findings indicated that TiW_11_Co-NS might be more effective in enhancing survival in mice than pure TiW_11_Co, suggesting its potential as a better therapeutic agent.

### 2.9. Computation Methods

#### Docking Studies

**Figure 9:**
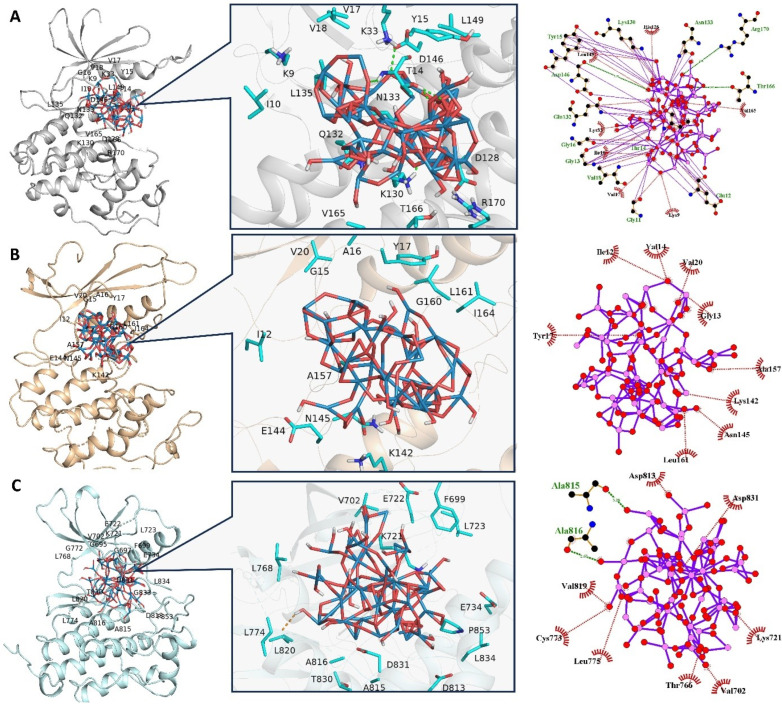
Generated docking complexes containing TiW_11_Co bonded to its corresponding molecular target. (**A**) CDK1-POM, (**B**) POM-CDK4, and (**C**) EGFR-POM. H-bonds are displayed in orange dotted lines.

Molecular docking is among the most employed approaches to unveil the binding mechanisms of ligands towards various molecular targets [45,46,47,48,49,50]. To elucidate the structural and functional dynamics inherent to ligand–protein interactions, the adoption of in silico methodologies offers substantial insights, particularly in understanding the molecular mechanisms that hinder the proliferation of cancer cells. This is exemplified through combined in vivo and in vitro analyses (as depicted in Figure 9A–C), where a marked diminution in the proliferation of MCF7 cell lines was observed after sustained exposure to POM. To deepen this understanding, a thorough review of existing literature was undertaken, pinpointing three critical proteins—Cyclin-Dependent Kinase 1 (CDK1), Cyclin-Dependent Kinase 4 (CDK4), and Epidermal Growth Factor Receptor (EGFR)—each playing a significant role in modulating distinct signaling cascades within MCF7 cells. This strategic identification of key proteins served as a cornerstone for further exploratory research into their intricate roles and interactions within the cellular milieu [51,52]. Subsequently, molecular docking was employed to unveil the mechanism underpinning the anti-proliferative potential of POM.

Utilizing the Consensus Score (cScore) as a metric, the most favorable ligand conformations within the active sites of the target proteins were delineated. The docking scores (cScore) for the inhibitor POM in its interactions with CDK1, CDK4, and EGFR were quantified as 48.32, 23.11, and 41.33, respectively. These values had underscored the formation of a particularly stable complex between POM and CDK1, alongside an efficient binding to EGFR. Conversely, POM exhibited a markedly lower binding affinity towards CDK4. Collectively, these docking scores had reflected a notable affinity of POM towards all three examined proteins, with a discernible tendency for CDK1. A comprehensive presentation of the docking results, inclusive of the scores, is provided in Table 3. To elucidate the observed variations in docking scores, the top-ranked docking complexes were preserved for detailed visual analysis (refer to Figure 9A–C). The significant conformational adaptations of the inhibitor POM, which assumed an intricate configuration post energy minimization. Despite POM’s complex architecture, rich in oxygen atoms, it manages to establish multiple hydrogen bond (H-bond) interactions with proximal residues in both CDK1 and EGFR complexes, corroborating its elevated docking scores. Particularly in the CDK1 complex, POM deeply infiltrates the active site, situated between the N- and C-terminal domains, engaging with residues including K9, G_11_, E12, G13, T14, Y15, G16, V18, K33, H126, K130, Q132, N133, D146, T166, and R170 (as depicted in Figure 9A). Notably, the H-bond interaction with the catalytic residue D146 is pivotal for the inhibition of CDK1. Intriguingly, the POM-CDK1 complex demonstrates a capacity to form an H-bond with D146. As illustrated in Figure 9A, the inhibitor POM predominantly interacted within the solvent-exposed and catalytic regions of CDK1. In the CDK1 complex, POM had established H-bond interactions with residues K33, Y15, D146, T166, and R170, with an average distance ranging approximately between 2.2 Å and 2.9 Å. These residues had been recurrently emphasized in prior research, underscoring their integral role in the binding dynamics of potent inhibitors to CDK isoforms, particularly CDK1 [53,54,55,56]. In the context of the CDK4 complex, POM was situated within the ligand-binding cavity, yet its extensive molecular structure impedes interaction with the hinge region. This spatial constraint was likely responsible for POM’s comparatively lower docking scores in the CDK4 system, as it fails to establish any hydrogen bond (H-bond) interactions within the CDK4 active site. Moving to the EGFR system, POM formed a limited number of H-bond contacts. The binding site for the EGFR-POM complex encompassed residues V702, K721, T766, C773, L775, D813, A815, D831, and A816. Mirroring the situation in the POM-CDK4 complex, POM in the EGFR-inhibitor complex predominantly localizes near the solvent-exposed region, not engaging the hinge residues. For an enhanced visual comprehension of these interactions, the 2D interaction diagrams of the inhibitor–protein complexes were elucidated in Figure 9A–C. Conclusively, these computational analyses shed light on the preferential binding affinity of POM towards CDK1. There was a strong congruence between these computational findings and the experimental data, presenting a cogent rationale for the significant chemo-preventive potential of POM against MCF7 cell lines.

## 3. Materials and Methods

This research study used materials obtained from Sigma Aldrich (St. Louis, MO, USA) and were utilized in their original form without any further purification, unless specified otherwise. These materials included hexa-potassium mono-hydrogen mono-titano-undeca-tungsto-cobaltate (II) mono-hydrate (referred to as TiW_11_Co), ethyl cellulose (EC), 100 kD, poly(vinyl alcohol) (PVA) 30 kD, acetic acid, dialysis membrane with a molecular weight cutoff (MWCO) of 10 kDa, dimethyl sulfoxide (DMSO), human breast cancer cell line (MCF–7, HTB-22^TM^) (ATCC, San Diego, CA, USA), human breast normal cell line (MCF-10A, CRL-10317) (ATCC, San Diego, CA, USA), Dulbecco’s modified Eagle’s medium, phosphate buffer (pH 7.4), trichloroacetic acid, tris-base, and Triton X-100.

### 3.1. Synthesis of NS

The emulsion solvent diffusion method was used to synthesize NS with varying concentrations of EC and PVA (see Table 1). Briefly, in this technique, the dispersed phase (organic phase) was prepared by ultrasonic stirring of EC (50–100% *w*/*v*) in 20 mL of ethanol. Then, PVA (50–100% *v*/*v*) and TiW_11_Co (1% *w*/*v*) were dissolved in 150 mL of water to prepare an aqueous continuous phase by stirring on a hot water bath at 60 °C. Afterward, dispersed phase was slowly incorporated in the continuous phase using syringe (5 mL, 22 G needle). This mixture was stirred at 10,000 rpm for 30 min with high-speed homogenizer (stalwart, SMT-30K Van Nuys, LA, CA, USA). To remove any adsorbed PVA, the TiW_11_Co-NS had been washed multiple times with ultra-pure water. Finally, the TiW_11_Co-NS were extracted by centrifugation (8000× *g*, 20 min) (Hettich EBA 200S, Singapore). The extracted NS were subjected to lyophilization by placing them in a glass vial and subjecting them to overnight at −40 °C in a single chamber LSCplus Martin Christ™ freezer in Germany. The lyophilized material was kept at 4 °C until the next round of experiments.

### 3.2. Entrapment Efficiency

The methodology that was formerly delineated endured only minor adjustments to calculate the entrapment efficiency (EE) percentage [57]. The TiW_11_Co-NS, weighing 657 mg (equivalent to 6.57 mg TiW_11_Co), was solubilized in 5 mL of phosphate-buffered saline (PBS) with a pH of 7.4. Subsequently, the solution was introduced into a dialysis membrane and subjected to agitation for one hour at a temperature of 37 °C using a magnetic stirrer operating at a speed of 100 rpm. At scheduled periods (0 and 1 h), a sample volume of 5 mL was drawn, and absorbance was recorded at a wavelength of 239 nm on a UV–visible spectrophotometer (Shimadzu UV 1900i, Tokyo, Japan) without any further dilution.

The % EE was estimated using the following equation:

$$EE\left(\%\right)=\frac{Total\;{TiW}_{11}Co\;added-{TiW}_{11}Co\;in\;supernatant}{Total\;{TiW}_{11}\;added}\times 100$$

### 3.3. Assessing Hydrodynamic Dimension and Zeta Potential: A Measurement Investigation

The TiW_11_Co-NS were dispersed in distilled ultrapure water to determine their hydrodynamic size. The Malvern Zetasizer Nano ZS (Cambridge, UK) was then used to examine the particle sizes and zeta potential [58].

### 3.4. Visual Examination Using Scanning Electron Microscopy (SEM)

The scanning electron microscopy (SEM) investigation was conducted using a Hitachi S-4700 (Houghton, MI, USA). The device was operated at an acceleration voltage that varied between 10 and 20 kV. The sample underwent fast dispersion in ethanol and then was deposited onto recently cleansed silicon wafers for the purpose of desiccation. In addition, a gold-sputter covering was used on the samples to enhance the conductivity of the material.

### 3.5. Exanimation Through Transmission Electron Microscopy (TEM)

The morphology of TiW_11_Co-NS (10 µL) was analyzed using transmission electron microscopy (TEM) using a JEM-2100 (JEOL; Tokyo, Japan) operated at a voltage of 200 kV. Prior to imaging, the samples underwent negative staining using a 2% *w*/*v* uranyl acetate solution, followed by a 20 min drying period. After the drying process, the samples were affixed onto a carbon-coated copper grid with a mesh size of 300 [59].

### 3.6. Release Kinetics of TiW_11_Co

The present work used a well-recognized methodology to examine the liberation of TiW_11_Co through NS with the aim of developing kinetic models [60]. In short, 657 mg TiW_11_Co-NS was dispersed in 5 mL of pH 7.4 phosphate-buffered saline. The mixture was placed on a dialysis membrane and immersed in 100 mL of pH 7.4 phosphate-buffered saline. A 75 rpm agitator kept the system at 37 °C. A UV–vis spectrophotometer (Shimadzu UV 1900i, Tokyo, Japan) at 239 nm measured TiW_11_Co release periodically. Using DDSolver software (an add-in program for Microsoft Excel (Xi’an Jiaotong University, Xi’an, China)), release kinetics-based mathematical models were used to determine the release process of pure TiW_11_Co from NS.

### 3.7. Stability Test of TiW_11_Co-NS

To examine the stability of TiW_11_Co inside NS, both the pure TiW_11_Co and TiW_11_Co-NS were kept in a controlled laboratory setting at a temperature of 25 °C for a duration of 6 months. The specimens were collected at certain time intervals (0, 60, and 180 days) and subjected to analysis using the UV–vis method (Shimadzu UV 1900i, Tokyo, Japan).

### 3.8. Enzyme Inhibition Analysis

A previously described absorbance method was used for enzyme inhibition investigation [61]. Briefly, TNAP was reconstituted in a pH 9.5 buffer solution with 50 mM Tris–HCl, 5 mM MgCl_2_, 0.1 mM ZnCl_2_, and 50% glycerol. The substrate, p–nitrophenyl phosphate (p–NPP), has been mixed in glycerol-free buffer. The assay commenced by combining enzyme (10 µL) with 10 µL of the test compound (both TiW_11_Co and TiW_11_Co-NS), followed by a 10-min pre-incubation period at 37 °C. Subsequently, the enzyme substrate (p–NPP) was added, and the mixture was further incubated for 30 min. Formation of the yellow-colored product (p–nitrophenolate) was observed, and absorbance was measured at 405 nm using an ELISA plate reader (BioTek ELx800^TM^, Instruments, Inc., Winooski, VT, USA). Each experiment was replicated three times, and the data were presented as IC_50_ values calculated using Prism 5.0 software (GraphPad Software, San Diego, CA, USA).

### 3.9. Pharmacological Assessment

#### 3.9.1. Sulforhodamine B Assay

The SRB assay was employed to assess and compare the anti-proliferative effects of pure TiW_11_Co and TiW_11_Co-NS on both MCF-7 and MCF-10A cells [62]. At the outset, 96-well plates were seeded with 1 × 10^4^ cells and given 24 h to multiply. Following this, at specified time intervals (24 h), different doses of pure TiW_11_Co and TiW_11_Co-NS were added to separate wells. Prior to attachment with 40% iced trichloroacetic acid (TCA), the cells were rinsed with PBS and allowed to air-dry after the incubation time. Following that, the cells were subjected to staining using a 0.4% *w*/*v* SRB dye for a duration of 30 min. Subsequently, a 100 µL (10 mM) solution of tris-base (pH 10.5) was administered. The microplate reader ELx808^TM^ (BioTek instruments, Winooski, VT, USA) was utilized to measure absorbance at 565 nm. IC_50_ values (µg/mL) were determined using Prism 5.0 software (GraphPad Software; San Diego, CA, USA).

#### 3.9.2. DAPI Staining Assessment

A two-well sterile chamber slide was used for cell culturing (1.5 × 10^4^). The cells were exposed to the test chemical (dose = IC_90_) for a duration of 24 h and then treated with a formaldehyde (4%) solution to fix them. The cells were subjected to a 5-minute incubation period with a solution of Triton X–100 (0.1%), followed by a subsequent washing with PBS. Afterwards, incubation of cells with DAPI (10 µg/mL) was carried out in the dark for a period of 10 min. Any unabsorbed DAPI was detached by washing with PBS several times, and cells were observed under a fluorescence microscope (Nikon Eclipse–Ni, Tokyo, Japan) at λ_emission_ 461 nm and λ_excitation_ 358 nm.

#### 3.9.3. Evaluation of Genotoxicity (Comet Test)

Quantification of DNA damage was conducted using the comet assessment [63]. MCF-7 cells (2 × 10^4^ cells/well) were treated with either pure TiW_11_Co or TiW_11_Co-NS. Prior to transfer onto comet slides, cultured cells were treated with 1% LMPA. Subsequently, the slides were submerged in an alkaline (10) lysis buffer amalgamated with Triton-X 100 and DMSO (1% and 0.1% *v*/*v* correspondingly) and 100 mM EDTA. Following this, the experiment proceeded with a time-course investigation, during which samples and buffer solution (consisting of 1 mM EDTA and 300 mM NaOH) at a pH of 13 were introduced into horizontally oriented electrophoretic-agarose chambers. An alkaline buffer was used to unwind the DNA. Following this, the glass slides were sanitized with CH_3_OH and thoroughly dried. The DNA extracted from the comet was analyzed using CaspLab 1.2.3b2 software.

#### 3.9.4. Analysis via Flow Cytometry

Following a 24-h exposure to pure TiW_11_Co and TiW_11_Co-NS, flow cytometry investigation was performed on the MCF-7 cell line (2 × 10^4^). The cells underwent trypsinization for a duration of 5 min at a temperature of 37 °C, using a mixture of trypsin and EDTA. To mitigate the formation of cell clusters, the cell culture medium was gradually introduced. Subsequently, cells were collected in 100 µL of binding buffer over a 15-min period after exposure to a concentration of 500 mM H_2_O_2_. Each cell was incubated for 15 min with the annexin-V FITC fluorescent marker and propidium iodide (PI) prior to being positioned in a less receptive environment. Using the proper FL2-A channel, analysis was carried out using fluorescence-triggered cell sorting (FACS) with wavelengths for emission of 600 nm for propidium iodide (PI) and 545 nm for annexin-V FITC. A comprehensive evaluation of 10,000 harvested cells was conducted in a singular iteration using the CytoFLEX equipment manufactured by Beckman-Coulter Life Sciences (Brea, CA, USA). The results were visually shown via a graph illustrating the examination of the cell cycle [64].

### 3.10. Studies Involving Animals

This study used mature female albino BALB/C mice with an average weight of roughly 30 g. The mice were housed at the animal facility located at Bahauddin Zakariya University in Multan, Pakistan. They were raised in controlled settings, which included a constant temperature of 25 °C, a 12-h light–dark cycle, and unrestricted access to food and water. Each steel-mesh cage housed up to five albino mice, ensuring ample space and minimizing stress due to overcrowding. In accordance with the guidelines set by the institution’s ethical committee (017/PEC/2023), the animals received humane housing and care. Randomly selected groups of five mice each were assigned to four experimental groups. Afterward, female mice were inoculated with roughly 4 × 10^6^ MCF-7 cells (100 µL). A duration of ten days was permitted for the tumors to grow to a volume ranging from 50 to 100 mm^3^. Specifics concerning the various groups of mice and the treatments they received are delineated in Table 4.

The tumor inhibition rate (%TIR) was utilized as a metric to evaluate the effectiveness of each formulation in combating cancer.

$$\%TIR=\left[\frac{Tumor\;weight\;in\;the\;experimental\;group}{Tumor\;weight\;in\;the\;reference\;group}\right]\times 100$$

Euthanasia had been performed as soon as an animal showed a rapid drop in body weight of more than 20 percent. The process was advised to be completed in a few hours, ideally on the same day, in situations where the symptoms had not been extremely severe but had yet satisfied the requirements for euthanasia to reduce prolonged and needless suffering. Timely detection and reaction had been ensured by constant observation.

Daily health and behavior observations were part of routine animal well-being monitoring to identify any major signs of weight loss, tumor development, or breathing problems. Throughout critical phases of the treatment, additional assessments were conducted to quickly act if the prerequisites for achieving the humane aim were met.

### 3.11. Computation Methods

#### Structures Preparation and Docking Studies

The three-dimensional conformation of TiW_11_Co was constructed using SYBYL-X1.3/SKETCH module [65]. For achieving a biologically active form, the POM structure underwent energy optimization via the Tripos force field to incorporate Gasteiger–Hückel atomic charges [66]. Co-crystal structures for Cyclin-Dependent Kinase 1 (CDK1), Protein Kinase B-alpha (PKB-α), and Epidermal Growth Factor Receptor (EGFR) were acquired from the RCSB Protein Data Bank, corresponding to PDB entries 4YC6 [67], 3G33 [68], and [69], respectively. These protein structures were processed using the integrated structure preparation tools of the SYBYL-X 1.3 Biopolymer module [70]. The Powell algorithm facilitated energy minimization, applying a convergence gradient of 0.5 kcal mol^−1^ across 1000 cycles. This procedure encompassed the addition of missing hydrogens, charging, and atom type assignment in accordance with the AMBER 7 FF99 force field [71]. Following this, the Surflex-Dock module in SYBYL-X 1.3 was employed to dock the energy-optimized, bioactive POM conformation into the active sites of CDK1, CDK4, and EGFR, replicating the methods and parameters from our previous studies [54,55]. The top twenty docking conformations were saved and analyzed for their binding interactions within the active sites of each target. These ligand poses were evaluated using the Hammerhead scoring function [65,72].

## 4. Conclusions

To summarize, the process of creating and analyzing nanosponges loaded with TiW_11_Co showed positive physical characteristics, stability, and increased capacity to kill cancer cells. Furthermore, in vitro investigations have shown enhanced inhibitory effects and apoptotic induction in comparison to TiW_11_Co in its pure form. The results were supported by in vivo tests, which demonstrated enhanced survival rates and tumor suppression after the injection of TiW_11_Co-NS. The process of molecular docking has provided more insight into the probable processes that contribute to the anticancer action of the compound, namely in its ability to target crucial proteins associated with cell cycle control and growth signaling.

The cytotoxic effects on tumor cells were boosted by the conversion of TiW_11_Co into hybrid TiW_11_Co-NS. This enhancement might be attributed to greater cellular uptake and specific delivery to the tumor location. Additional investigation might be necessary to clarify the processes that contribute to the increased effectiveness of TiW_11_Co-NS, as well as to optimize the dose and treatment protocols to achieve the highest possible therapeutic advantage. Furthermore, it is recommended that future research endeavors delve into the safety characteristics and possible adverse reactions of TiW_11_Co-NS in vivo to ascertain its appropriateness for clinical use.

## Figures and Tables

**Table 1:** Physical characteristics of NS at different concentrations of EC and PVA, with a constant TiW_11_Co percentage of 1% *w*/*v* (mean ± SD, n = 3).

	F1	F2	F3	F4
TiW_11_Co (% *w*/*v*)	1	1	1	1
EC (% *w*/*v*)	50	50	100	100
PVA (% *w*/*v*)	50	100	50	100
rpm	10,000	10,000	10,000	10,000
Particle size mean ± SD (nm)	126 ± 4	109.5 ± 8	471 ± 6	324 ± 7
Loading efficiency	23.48 ± 7.19	34.25 ± 4.37	40.57 ± 5.64	42.74 ± 6.02
Entrapment efficiency	75.11 ± 6.61	82.16 ± 5.91	78.32 ± 6.18	85.9 ± 3.21
PDI mean ± SD	0.095 ± 0.0027	0.031 ± 0.0094	0.104 ± 0.083	0.136 ± 0.098
Zeta potential mean ± SD (mV)	−24.91 ± 6.21	−27.08 ± 3.79	−18.57 ± 4.05	−20.34 ± 2.18

**Table 2:** Exploration of multiple variables of comet assay treated with TiW_11_Co-NS and pure TiW_11_Co on the MCF-7 cell line.

Parameters of Comet Assay	Pure TiW_11_Co	TiW_11_Co-NS
L-Head	79	113
L-Tail	21	63
L-Comet	108	176
Head-DNA	90.41	71.01
Tail-DNA	5.38	19.14
TM	0.779	11.67
OTM	3.16	10.11

**Table 3:** The Surflex scores were calculated for the docked ligand POM within the binding sites of proteins CDK1, CDK4, and EGFR.

Inhibitor.	Docking Complex	CScore ^a^	Crash Score ^b^	Polar Score ^c^	D Score ^d^	PMF Score ^e^	G Score ^f^	Chem Score ^g^
POM	CDK1	48.32	−10.43	36.34	−5654.41	−110.74	−1043.13	−35.64
	CDK4	23.11	−3.13	66.82	−1259.23	−86.34	−436.87	−12.64
	EGFR	41.33	−8.55	44.14	−1985.35	−98.68	−876.65	−17.95

Utilizing a variety of evaluation methods, ^a^ CScore determines the compatibility between ligands and their respective binding sites. ^b^ Crash score acts as a vigilant guard, identifying instances of unwarranted intrusion into the binding regions. ^c^ Attention is paid to the polar characteristics of ligands. ^d^ D score meticulously scrutinizes the interplay of hydrogen bonds, as well as the intricate dance of energies within and between ligand–protein and ligand–ligand complexes. ^e^ PMF score delves into the realm of Helmholtz free energies, dissecting the interactions between individual protein–ligand atom pairs. ^f^ G score captures the essence of charge dynamics and van der Waals forces governing the interplay between proteins and their bound ligands. ^g^ Chem score, with its nuanced approach, rewards instances of hydrogen bonding, lipophilic contacts, and the subtle dance of rotational entropy, all while incorporating an intercept term for added depth.

**Table 4:** Animals in control and experimental groups administered pure TiW_11_Co, TiW_11_Co-NS, and cisplatin.

Group	Type of Treatment
1	Mice that did not receive any treatment.
2	The cancer-stricken mice were given cisplatin 9.99 µM/kg (dose = IC_90_)
3	The cancer-stricken mice were given pure TiW_11_Co (4.41 µM/kg) (dose = IC_90_)
4	The cancer-stricken mice were given TiW_11_Co-NS (1.756 µM/kg) (dose = IC_90_)

## Data Availability

The data and material generated during the current study are available upon reasonable request from the corresponding author.
